# Pennsylvania Hospital

**Published:** 1840-04-01

**Authors:** 


### PENNSYLVANIA HOSPITAL.

### REPORT ON INJURIES OF THE HEAD.*

THE Report in our American contemporary from which we take the following cases, is by Dr. Kirkbride, a gentleman who frequently lays before the public the most interesting cases in this Institution.

Injuries of the Head are so varied in their character, and so uncertain in their results, that the cases can scarcely be too much multiplied, or the data, from our reasoning and our practice too ample. We seldom, therefore, lose an opportunity of noticing such cases as seem to carry instruction along with them.

CASE 1. *Compression of the Brain from large Effusion of Blood—Rupture of the Middle Artery of the Dura Mater, without Fracture of the Skull—Death.*—A.C.M., ætat. 18, while engaged at his occupation as a carpenter, fell from the third story of a house on the 15th of June, 1833. He alighted directly upon his head, in a fire-place in which were small pieces of brick. He was rendered completely insensible by the fall, and was taken to the Pennsylvania Hospital, forty minutes after the occurrence of the accident. At that time his skin was warm—pulse full and strong—his face flushed—pupils dilated—breathing stertorous—several small incisions existed in the scalp, produced by the rough bodies upon which he had fallen, but no evidence of fracture or depression in any part of the cranium. A vein was opened in the arm, and until twelve ounces of blood were drawn, no change took place in the pulse; it then began to sink and the aperture was immediately closed. He died thirty minutes afterwards.

*Dissection, six hours after death.*—All the upper portion of the scalp, in which were found the wounds mentioned above, was elevated by the effusion of full half a pint of blood between it and the bone. Beneath the cranium, and between it and the dura mater was found ℥viij. of fluid blood. Under the membranes, in the ventricles, and at the base of the brain was not less than ℥xvj. of effused blood, also in a fluid state. The middle artery of the dura mater was ruptured.

CASE 2. *Concussion—Bleeding from the Ear and Nose—Convulsions—Recovery.*—L. T., aged 6, fell at 8 p.m. July 12, 1838, a distance of five or six feet, striking his head upon a block of wood lying in a cellar. He was rendered insensible by the fall, but spoke some minutes afterwards. He was bled twenty

---

* The American Journal of Medical Sciences, Nov. 1839.

minutes after the accident to the extent of eight or nine ounces; after which he appeared prostrated—vomited, and had a convulsion.

9 p.m. There was a contusion and swelling over the left parietal protuberance—but no wound of the scalp, nor could any fracture or depression of bone be detected. His pulse was feeble, 100; skin cool and pallid; his pupils were somewhat dilated; he had bleeding from the ear and nose; swallowed drink, and although he did not speak, appeared to recognise his father when addressed by him. During the visit he vomited his undigested supper, mixed with blood, and soon after had a violent convulsion. His extremities were rigid, and spasmodic action of the eye-lids and mouth continued for several minutes. His respiration at one time appeared almost stertorous, and the pupils of his eyes were, for a period, noticed to be strongly contracted. A stimulating enema was given—sinapisms applied over the stomach and on the legs, with heat to the extremities and ice to his head. After this he slept soundly, but seemed to feel cold on the head. At 11 p.m. the pulse was 100, quick, with more strength; skin warm; pupils contracted. ℞. Calomel gr. vj.; Mist. neutral. with antim. tartrat. Ice continued to head.

The patient slept that night, but could be roused, and vomited several times after drinking. On the 13th, the pulse 90—pupils natural—skin warm—bowels not open. *A stimulating enema, ice and antimonial mixture to be continued.*

14th. Can converse—some irritability of stomach—scarce any fever.

No further symptom. On the 21st he was discharged. He afterwards remained well.

### Cases of Compound Fracture of the Cranium.

1. *Compound Fracture of the Skull, with Depression—Recovery, without an Operation.*—W. R. aged 9, at 7 P. M. June 24, 1836, fell 8 ft. striking the right side of his head upon the edge of an iron furnace. Some minutes afterwards, he was heard to cry, which alarmed his family. In an hour, Dr. Kirkbride saw him in consultation with Dr. Perrine. He then was rational and answered questions properly. He vomited the undigested contents of his stomach—complained of the pain of the wound, and resisted strongly while his head was being shaved. His pulse 80, regular, rather quick and weak—pupils natural—he was disposed to dose, but was always roused without difficulty.

On the right side of the head, two inches from the mastoid process, anteriorly and superiorly, was a wound, three-fourths of an inch in extent, from which blood continued to ooze. Upon separating the lips of this wound, a portion of denuded bone was observed, and a fissure with depression of a fragment of bone on its lower side. With the finger, this depressed portion was readily traced, it was one inch and three quarters long and nearly as wide;—the depression at the fissure, was of the thickness of the bone, and at the other edge was on a level with the service of the cranium. A small wound also existed near the lower edge of the fragment, but it did not penetrate to the bone. As there were no symptoms whatever denoting compression of the brain, it was determined to resort to a rigorously antiphlogistic treatment and not to operate, at least, until the occurrence of other indications for that course. *V.S. ad* ℥vj—*calomel purge—ice.*

Next day, the pulse was 80, and until the 27th, he may be said to have had no symptoms. On that day, there was more tenderness around the wound, and twenty leeches were applied. On the 4th July, he was out of bed—on the 11th, the wound was healed. He continued well afterwards.

2. *Compound Fracture of the Skull, with loss of Cerebral Substance—Rationality for several days—Death from Phrenitis.*

J. H. aged 28, a labourer, admitted Jan. 16, 1837, at 3 P. M. At 11 o'clock the same morning, he had received a kick from a horse upon the frontal bone immediately above the right eye. He was stunned for a short time—had considerable hæmorrhage, but no vomiting. Upon his admission his skin was cold and pulse feeble; he was rational, spoke of the occurrence of the accident, and noticed remarks made upon the severity of the injury. Small pieces of bone were removed through the opening of the scalp, which was nearly two inches in extent, and through this a much larger portion of bone appeared to have escaped before he reached the hospital; portions of the cerebral substance had passed through the torn dura mater, and the finger could be introduced without difficulty into the cerebrum. The eye of the injured side could not be seen from the great effusion which prevented its being opened; the pupil of the opposite side was natural; there was no distortion of the features, nor paralysis of the extremities.

Next day, the face was a little flushed, and he had headache; the pulse 60. In the night of the 18th, he was restless and would walk about the ward. On the 19th, his pulse was 65. He recollected all.

*20th.* More restless; tossing about the bed; pulse quick, 90; face flushed; skin hot and dry; tongue covered with a whitish fur, and inclined to dryness; the pupil of the left eye is natural, but the expression is changed; pain in the head more severe.

*21st.* Very restless during the night and delirious; pulse 120, quick and feeble; skin cool, inclined to moisture; respiration 40; sanious discharge from the wound; pupils slightly dilated; no paralysis.

After this he gradually sunk, and died at eight P. M. of the 23rd.

*Dissection.—Head only examined.*—The right orbital plate of the frontal bone broken up, and portions of it still upon or imbedded in the brain; the ethmoid, and the wing of the sphenoid also fractured; the zygomatic process of the temporal bone broken and loose. Two openings, admitting the finger, existed in the dura mater, and a fragment of bone three-fourths of an inch long was found nearly one inch from the surface of the cerebrum; four other pieces of nearly the same size were also found in the substance of the brain or immediately upon it at some distance from the softened portion. On the *right* side, both surfaces of the arachnoid were covered with a tenacious pus, so as to completely hide their proper surface. This coating extended to the posterior part of the cerebrum. No pus in the ventricles, which contained two ounces of fluid. The brain, at the seat of the injury, two and a half inches in extent, by one and a half in depth, was softened so as to form a bloody pultaceous mass. The pia mater injected; scarce more than the usual quantity of red points in other portions of the cerebrum. On the *left* side the membranes and substance of the brain were nearly normal in their appearance.

3. *Epilepsy after the use of the Trephine for Compound Fracture of the Skull.* R. B., aged 45, had resided in the State of Ohio for a long period, and fourteen years since was injured by a tree falling upon him and fracturing his skull so severely, that he underwent the operation of trephining, and had a number of pieces of bone removed, one of which was one and a half inches by three-fourths of an inch in extent. This space is filled up by ligament, and there exists a depression so striking as to arrest attention, upon the most careless examination of his head. He recovered from the operation in a few weeks, but since that time has been subject to epileptic fits, although his health in other respects is good. The fits recur at irregular intervals, but come on with great certainty after an unusual indulgence in drink.

Dr. Kirkbride, in some observations which he appends to these cases, observations which have chiefly reference to the use of the trephine, laments the discrepancies of opinion that still obtain with regard to it, and attributes them in some degree to surgeons trusting more to their memory than to facts.

